# Parapatric speciation in three islands: dynamics of geographical configuration of allele sharing

**DOI:** 10.1098/rsos.160819

**Published:** 2017-02-22

**Authors:** Ryo Yamaguchi, Yoh Iwasa

**Affiliations:** Department of Biology, Faculty of Science, Kyushu University, 744 Motooka, Nishi-ku, Fukuoka 819-0395, Japan

**Keywords:** genetic drift, neutral loci, recurrent migration, speciation rate, stochastic model

## Abstract

We studied the time to speciation by geographical isolation for a species living on three islands connected by rare migration. We assumed that incompatibility was controlled by a number of quantitative loci and that individuals differing in loci by more than a threshold did not mix genetically with each other. For each locus, we defined the geographical configuration (GC), which specifies islands with common alleles, and traced the stochastic transitions between different GCs. From these results, we calculated the changes in genetic distances. As a single migration event provides an opportunity for transitions in multiple loci, the GCs of different loci are correlated, which can be evaluated by constructing the stochastic differential equations of the number of loci with different GCs. Our model showed that the low number of incompatibility loci facilitates parapatric speciation and that migrants arriving as a group shorten the waiting time to speciation compared with the same number of migrants arriving individually. We also discuss how speciation rate changes with geographical structure.

## 1. Introduction

Speciation by geographical isolation is an important means by which new species are created [1–3]. If a species is subdivided into a number of subpopulations, they will accumulate novel mutations and will eventually become very different from each other. When individuals from different populations mix, they may no longer be able to engage in sexual reproduction. The two populations can then be regarded as different species produced via allopatric speciation (if migration does not occur) or parapatric speciation (if migration occurs at a low rate). Although this process is widely recognized as a major form of species origination [4,5], the theoretical study of the process of allopatric/parapatric speciation, particularly for more than two populations, has not been widely examined in the last several decades [6,7]. However, most theoretical studies of the speciation process have focused on the plausibility and generality of sympatric speciation, in which species diverge by adapting to different habitats or niches or by evolving different mate choices [8–16].

A classical framework proposed by Dobzhansky [17] and Muller [18] was a simple two-locus two-allele diploid model describing the evolution of genetic incompatibility between two populations. Suppose that the initial genotype was aabb in both populations. In one population, this genotype was replaced by AAbb by neutral evolution because aabb, Aabb and AAbb have the same fitness. In the other population, aabb was replaced by aaBB by neutral evolution. Because their hybrid AaBb is not viable, the two populations are no longer able to mix with each other. An even simpler model of allopatric speciation considering a single locus with multiple alleles has also been developed [19,20]. Some theoretical studies on allopatric speciation have examined various extensions of these models[21–24]. These studies examined, for example, how the time to speciation is affected by population size [25] or the founder effect [26]. In addition, from a review of published literature, Coyne & Orr [3,27] concluded that the number of loci controlling the incompatibility varies substantially between species and between focal traits (viability, sterility, male courtship, sexual traits, pheromone, etc.). In many cases of putative recent speciation, often more than two loci and sometimes more than 10 loci are involved in reproductive isolation.

In many of these theoretical studies, it was assumed that no migration occurred among populations when calculating the time to speciation. However, empirical studies have suggested that the most common mode of speciation is parapatric: different populations are indeed isolated, but not absolutely [28,29]. The geographical structures of most species are consistent with the existence of meta-populations composed of many local populations connected by infrequent migration [30,31]. Gavrilets [32] studied a model of parapatric speciation incorporating recurrent migration between two populations. As an extension from two loci to multiple loci, when incompatibility is controlled by a number of loci, the two populations become separate species when the number of incompatibility-controlling loci different between populations exceeds a threshold value. Assuming that each population is monomorphic, he analysed the birth-and-death process and found that the waiting time for speciation increases with the migration rate between populations. Using the diffusion approximation, Yamaguchi & Iwasa [33] developed analytical predictions for the time to speciation and found that the rate of species origination by recurrent parapatric speciation is maximized at an intermediate migration rate. Yamaguchi & Iwasa [34] focused on stochasticity created by finiteness of the number of incompatibility loci and concluded that the low number of incompatibility-controlling loci considerably shortens the waiting time to speciation.

All of these studies of parapatric speciation focused on cases with two islands (e.g. [32–34]). In this study, we examined parapatric speciation when there are three populations. Specifically, we consider the time until the genetic difference of two out of three islands becomes so large that the species can no longer genetically mix with each other. A single migration event between islands provides an opportunity for multiple loci to replace a resident allele with a migrant sequence. This causes correlation between loci. The mean of the changes in genetic distances between populations are rather simple to determine, but the calculation of variance and covariance of the changes is very difficult. This problem is absent when there are only two islands. In this study, in order to overcome this difficulty, we introduce a new method of calculation. We defined the geographical configuration (GC) for each locus and trace the transition of loci between different configurations. We derived the stochastic dynamics of the fraction of loci with different GCs, and then calculated genetic distances between islands from GCs of loci.

## 2. Material and methods

### 2.1. Two populations: dynamics of genetic distance

Before considering the problem of the three islands, we briefly summarize the formalism and behaviour of the model with two islands [33,34]. For simplicity, we consider a sexually haploid species with discrete, non-overlapping generations living on two islands, each with a population size of *N*. Migration between the islands occurs only infrequently because the populations are distant from each other. When a migration attempt is successful, some number of individuals from one population arrive as a group and participate in reproduction. We assume that the mating compatibility between individuals is controlled by a set of *l* autosomal loci. Specifically, the possibility of successful mating between immigrants and residents is determined by the fraction of loci different between them, which is denoted by *z*. Two individuals cannot produce viable and fecund offspring if *z* exceeds a threshold value, *z_c_*. By contrast, individuals can mate and produce fully viable offspring if *z* is less than *z_c_*. We define the genetic distance as the fraction of incompatibility-controlling loci for which two populations are different.

We assumed that the size of each population *N* is markedly smaller than the inverses of the overall mutation rate *ul* of the incompatibility-controlling loci and the migration rate (1/ul≫N and 1/m≫N). This assumption renders island populations monomorphic most of the time, except for brief periods of allelic replacement and migration events [35–42]. We trace the stochastic changes in the value of *z* over time. Genetic difference between two islands increases because of accumulation of novel mutations, and decreases after migration and subsequent hybridization.

The model we propose in this paper describes the stochastic dynamics of the genetic distance, which are controlled by rare migration and by mutation accumulation. Note that our dynamics focuses on a time scale much longer than the stochastic dynamics of population genetics, which traces the change in gene frequencies. Hence in our dynamics, the fixation or extinction of novel mutations or the fixation after migration events occur instantly. To keep this assumption, the population size should be small enough.

### 2.2. Change in genetic difference

Mutation and replacement on one island occurs at rate *u*. Since there are *l* loci, the average rate of allelic replacement on one island is *ul*. When allelic replacement occurs at a locus, the genetic distance increases only if the two populations shared the same allele before replacement. Thus, the increase in *z* over a short time interval Δ*t* because of this allelic replacement on island A is given by

2.1 $${\left(\Delta z\right)}_{A}^{\mathrm{rep}}=\left\{ \begin{array}{ll} \frac{1}{l}, & \mathrm{with}\;\mathrm{probability}\;ul(1-z)\Delta t,\\ 0, & \mathrm{with}\;\mathrm{probability}\;1-ul(1-z)\Delta t. \end{array}\right.$$

The mutation and replacement on island B also cause a similar increase in the genetic distance at rate *u*, which is denoted by ${\left(\Delta z\right)}_{B}^{\mathrm{rep}}$.

The timing of a successful migration event from one population to the other follows a Poisson point process. The rate is *m* per generation, and *m* is very small because the interval between successful migrations is very long. If the fraction of loci different between immigrants and residents is greater than the threshold value *z_c_*, the immigrants and residents do not mix sexually and should be treated as different species. By contrast, if *z* is smaller than the threshold *z_c_*, individuals from the two populations can freely exchange genomes via sexual reproduction. The stochastic theory of population genetics (as applied to neutral loci) indicates that a population eventually becomes monomorphic when one allele becomes fixed and the other becomes extinct, and the probabilities of the fixation of two alleles are proportional to their initial frequencies [43,44]. If the population size of immigrants is *N*′ and that of residents is *N*, immediately after migration the population is polymorphic at *lz* loci. Based on the inequality m≪1/N, an allele(s) introduced by a migration event either becomes fixed or is lost before the next migration event occurs. After a certain number of generations, which is in the order of *N*, the population again becomes monomorphic, and the expected fraction of loci carrying immigrant alleles equals the fraction of immigrants: $\varepsilon =N^{\prime }/(N+N^{\prime })$. Here we handle situations in which migrant population size is much smaller than the whole population size (ε≪1). For simplicity, we assume that the loci are unlinked. If the loci behave independently (i.e. free recombination assumption), the frequency of such loci that still differ after reproduction follows a binomial distribution *B*(*lz*, *ε*). Therefore, the decrease in *z*(*t*) occurring over a short time interval Δ*t* is given by

2.2 $${\left(\Delta z\right)}_{\mathrm{AB}}^{\mathrm{mig}}=\left\{ \begin{array}{ll} \frac{-i}{l}, & \mathrm{with}\;\mathrm{probability}\;m\left(\begin{array}{c} lz\\ i \end{array}\right)\varepsilon^{i}{\left(1-\varepsilon \right)}^{lz-i}\Delta t,\;(i=0,1,\ldots ,lz),\\ 0, & \mathrm{with}\;\mathrm{probability}\;1-m\sum_{i=0}^{lz}\left(\begin{array}{c} lz,\\ i \end{array}\right)\varepsilon^{i}{\left(1-\varepsilon \right)}^{lz-i}\Delta t. \end{array}\right.$$

The migration from island B to island A also occurs at rate *m* and results in a similar reduction of distance, denoted by ${\left(\Delta z\right)}_{\mathrm{BA}}^{\mathrm{mig}}$. It also follows equation (2.2).

### 2.3. Stochastic differential equation

The change in *z* within a short time interval of length Δ*t* is given by

2.3 $$\Delta z=M(z)\Delta t+\sqrt{V(z)}\cdot \Delta W,$$

where Δ*W* is a Gaussian random variable with a mean of zero and variance Δ*t* and is independent of the time interval considered. Two functions of *z* on the r.h.s. of equation (2.3) indicate the mean rate of increase in *z*, $M(z)=\underset{\Delta t\to 0}{\lim}E[\Delta z|z]/\Delta t$ and the rate of variance generation $V(z)=\lim_{\Delta t\to 0}\mathrm{Var}[\Delta z|z]/\Delta t$. Equation (2.3) is a stochastic differential equation (SDE) and can be used for numerical simulation of stochastic processes of *z* (see the electronic supplementary material, S1 Text). This equation includes only the mean change and the variance of the change in the focal variable. In the limit when the length of interval Δ*t* is short, moments higher than the second order would not contribute to the behaviour of the model (e.g. [45,46]).

As the change in variables over a short time period Δ*t* is small, the diffusion approximation is maintained. We can use diffusion approximation for the condition that many loci control a reproductive trait. This assumption allows us to approximate *z* as a continuous variable. According to probability theory, any stochastic process with continuous time and continuous space is a diffusion process if it is Markovian and if the trajectory is continuous with probability one. Thus, one can focus on the mean, variance and covariance of changes Δ*z*. In population genetics, the pseudo-sampling method (cf. [47]) has been used for checking the results obtained by diffusion equation. This Monte Carlo experiment is mathematically equal to our SDE method.

Using equation (2.1), we can calculate the change in *z* caused by replacement of a sequence in population A as follows:

2.4 $${\left(\Delta z\right)}_{A}^{\mathrm{rep}}=u(1-z)\Delta t+\sqrt{\frac{u(1-z)}{l}}\cdot \Delta W_{A}^{\mathrm{rep}}$$

(see the electronic supplementary material, S1 Text, for derivation). We can derive a similar formula, ${\left(\Delta z\right)}_{B}^{\mathrm{rep}}$, indicating the change in *z* when replacement occurs on island B (electronic supplementary material, S1 Text). Based on equation (2.3), the change in *z* caused by migration occurring from island A to island B and the subsequent fixation of an invader's allele in some loci is described as

2.5 $${\left(\Delta z\right)}_{\mathrm{AB}}^{\mathrm{mig}}=-m\varepsilon z\Delta t+\sqrt{m}\varepsilon z\cdot \Delta W_{\mathrm{AB}}^{\mathrm{mig}}+\sqrt{\frac{mz\varepsilon (1-\varepsilon )}{l}\cdot }\Delta W_{\mathrm{AB}}^{\mathrm{fix}}.$$

The first and second terms on the r.h.s. indicate Poisson point processes at rate *m*, each causing change (−1)*εz*. The third term is represented by the binomial probability distribution, describing the variance associated with the sampling of migrant alleles that reached fixation. In a similar manner, we can derive the corresponding formula for the change caused by migration from B to A, denoted by ${\left(\Delta z\right)}_{\mathrm{BA}}^{\mathrm{mig}}$ (see the electronic supplementary material, S1 Text).

### 2.4. Case of three islands: dynamics of geographical configurations

When there are only two populations, (AB) indicates that both populations have the same allele, while (A)(B) indicates that the populations differ. We denote their fractions as 1 − *z* and *z*, respectively. Hence, the genetic distance between two islands is equal to *z*, which is the fraction of loci with GC (A)(B).

By contrast, the fractions of GCs are not equivalent to the genetic distances when there are three islands. There are five GCs: (ABC), (A)(BC), (B)(CA), (C)(AB) and (A)(B)(C). Let *z*_1_ be the fraction of loci of GC (ABC) and *z*_2_, *z*_3_, *z*_4_ and *z*_5_ the numbers of loci of status (A)(BC), (B)(CA), (C)(AB) and (A)(B)(C), respectively. The sum of these five fractions is equal to unity: $\Sigma_{i=1}^{5}z_{i}=1$. Since their sum is equal to unity, specifying fractions of loci has four degrees of freedom. By contrast, there are only three genetic distances between pairs of islands. Once we know the fraction of loci with different GCs, we can calculate the genetic distances between three pairs of islands as follows:

2.6 $$z_{\mathrm{AB}}=z_{2}+z_{3}+z_{5},\;z_{\mathrm{BC}}=z_{3}+z_{4}+z_{5}\;\mathrm{and}\;z_{\mathrm{CA}}=z_{2}+z_{4}+z_{5}.$$

However, the opposite does not hold; even if we are given three genetic distances between three pairs of islands, we cannot specify the fraction of GCs. There are different fractions of GCs that give exactly the same genetic distances among islands. Suppose one-third of *l* loci have (ABC) and two-thirds of *l* loci have (A)(B)(C). The genetic distances between AB, between BC and between CA are all 2/3 ($z_{\mathrm{AB}}=z_{\mathrm{BC}}=z_{\mathrm{CA}}=2/3$). Let us call this situation case 1. We consider case 2 in which loci with (A)(BC), with (B)(CA), and with (C)(AB) are all equally abundant, each accounting for one-third. In case 2, we have $z_{\mathrm{AB}}=z_{\mathrm{BC}}=z_{\mathrm{CA}}=2/3$ again.

For a migration event, the incompatibility between migrants and residents is determined by the genetic distance between them. We aim to predict the distance between pairs of islands. We can show that the stochastic changes in genetic distances among the three islands differed from the GCs satisfying the same genetic distances. Consider case 1 and case 2 explained above. The genetic distances between three islands are the same between two cases. Suppose that successful migration occurs from island A to island B, and consider the subsequent change in genetic distances between islands by hybridization. The changes in *z*_AB_ and *z*_CA_ are the same between case 1 and case 2. However, according to the calculation shown in the electronic supplementary material, S2 Text, the change in *z*_BC_ differs between the two cases: the variance of Δ*z*_BC_ is zero in case 1 but zε(1 − *ε*)/*l* in case 2. Based on our experience with the two-island case explained in the previous section, the variance of genetic distance is extremely important for estimating the time to speciation. Hence, we cannot form stochastic dynamics for three genetic distances only. Instead, we need to construct the stochastic dynamics of five GCs, from which we can calculate the genetic distances.

*z_i_* may change upon accumulation of novel mutations on different islands and upon migration among islands. As the change over a short period Δ*t* is assumed to be small, we can approximate such changes as follows:

2.7 $$\Delta z_{i}={\left(\Delta z_{i}\right)}_{\mathrm{accumulation}}+{\left(\Delta z_{i}\right)}_{\mathrm{migration}},\;i=1,2,\ldots ,5,$$

where ${\left(\Delta z_{i}\right)}_{\mathrm{accumulation}}$ is the change caused by the accumulation of novel mutations on one island, and ${\left(\Delta z_{i}\right)}_{\mathrm{migration}}$ is that caused by successful migration events, i.e. migration events that lead to the fixation of the migrant allele. Thus, one can focus on the mean, variance, and covariance of changes Δ*z_i_*. Below, we calculate these quantities.

### 2.5. Mutation and allelic replacement

**Figure 1.**
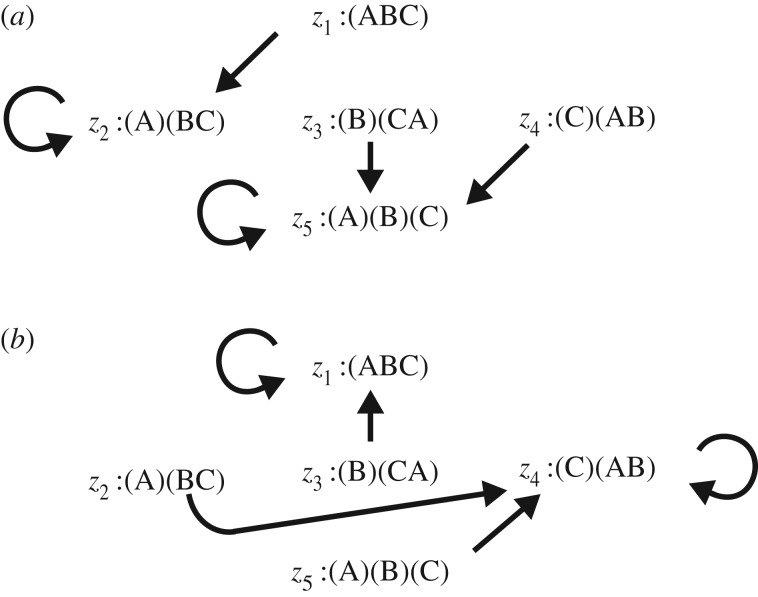
Transitions among configurations in the three-island model. (*a*) When a novel mutation occurs and becomes fixed on island A. Each arrow indicates a transition from one configuration to the next caused by this fixation. For example, (ABC) indicates that three populations share a common allele at the focal locus. When replacement of an allele occurs in population A, the configuration becomes (A)(BC), which indicates that the population on islands B and C are the same, but the population on island A is different. This is indicated by an arrow from (ABC) to (A)(BC). (*b*) When a successful migration event occurs from island A to island B, and the invader allele becomes fixed in the population on island B. Suppose that before the migration event, the focal locus has configuration (B)(CA), indicating that islands A and C have the same allele. After migration and fixation of the invader allele, population in B becomes the same as population A. Thus, all three islands now have a common allele, as indicated by the configuration (ABC). This is indicated by an arrow from (B)(CA) to (ABC). Note that different loci may experience the transition independently from each other, and also that a single migration event allows different loci to transition simultaneously. See the explanation in the text.

We first consider the situation in which a novel mutation occurs on an island and replaces the resident allele (figure 1*a*). Let Δ*t* be a short time interval, in which the numbers of loci differing in configuration do not change appreciably. As an example, we consider the change in *z*_2_, the fraction of loci with configuration (A)(BC), indicating that islands B and C share the same allele which differ from the fixed allele on island A. Assume that the original configuration was (ABC), indicating that all three islands share the same allele. If allele replacement occurs on island A, the GC changes from (ABC) to (A)(BC), which increases *z*_2_. However, if the original configuration was (A)(BC) and if allele replacement occurs either on island B or C, the GC (A)(BC) changes to (A)(B)(C) and *z*_2_ decreases, which can be represented as follows:

2.8 $${\left(\Delta z_{2}\right)}_{\mathrm{accumulation}}=\left(uz_{1}\Delta t+\sqrt{\frac{uz_{1}}{l}}\Delta W_{p}\right)-\left(uz_{2}\Delta t+\sqrt{\frac{uz_{2}}{l}}\Delta W_{q}\right)-\left(uz_{2}\Delta t+\sqrt{\frac{uz_{2}}{l}}\Delta W_{r}\right),$$

where Δ*W_p_*, Δ*W_q_* andΔ*W_r_* are independent Gaussian random variables with means of zero and variances Δ*t*. The first bracketed term on the right of equation (2.8) represents the change in *z*_2_ caused by allele replacement on island A when the original configuration was *z*_1_. Since the event exhibits a Poisson distribution, the variance corresponds to the mean. The second and the third bracketed terms on the right of equation (2.8) represent the changes in *z*_2_ caused by allele replacement on islands B and C, respectively, when the original configuration was *z*_2_.

Similarly, we can generate equations giving the fractions of loci with five GCs (*z_i_*, *i* = 1, 2, … ,5). Let *g*(*α*,*i*) be the number of loci that originally had the GC *i* and that accumulated novel mutations on island *α* (*α* = A, B, C) over the time Δ*t*:

2.9 $$g(\alpha ,i)=uz_{i}\Delta t+\sqrt{\frac{uz_{i}}{l}}\Delta W_{i}^{\alpha },$$

where $\Delta W_{i}^{\alpha }$ are independent Gaussian random variables with means of zero and variances Δ*t*. The following equations give the changes in the frequencies of loci of different GCs caused by the accumulation of novel mutations:

2.10*a* $$\begin{array}{rl} {\left(\Delta z_{1}\right)}_{\mathrm{accumulation}} & =-g(A,1)-g(B,1)-g(C,1), \end{array}$$

2.10*b* $$\begin{array}{rl} {\left(\Delta z_{2}\right)}_{\mathrm{accumulation}} & =g(A,1)-g(B,2)-g(C,2), \end{array}$$

2.10*c* $$\begin{array}{rl} {\left(\Delta z_{3}\right)}_{\mathrm{accumulation}} & =g(B,1)-g(A,3)-g(C,3), \end{array}$$

2.10*d* $$\begin{array}{rl} {\left(\Delta z_{4}\right)}_{\mathrm{accumulation}} & =g(C,1)-g(A,4)-g(B,4) \end{array}$$

2.10*e* $$\begin{array}{rl} \;\mathrm{and}\;{\left(\Delta z_{5}\right)}_{\mathrm{accumulation}} & =g(A,3)+g(A,4)+g(B,2)+g(B,4)+g(C,2)+g(C,3). \end{array}$$

Equation (2.10*b*) corresponds to equation (2.8). Note that some of the transitions in equations (2.10*a*–*e*) are independent, while others are not. For example, the first term on the right of the first equation must be the same as the first term of the second equation.

### 2.6. Migration and subsequent hybridization

Next, we consider transitions between GCs caused by migration among islands. Successful migration of a group of individuals from island A to island B occurs at a rate of *m* per generation. Following successful migration, the GC may change as the population genetic process varies (figure 1*b*). For example, if a migrant allele becomes fixed, configuration (A)(BC) changes to (C)(AB). If, instead, a migrant allele becomes extinct, the configuration remains (A)(BC).

We first consider the stochastic process whereby *z_i_* changes over a time of length Δ*t*. The number of successful migration events in Δ*t*, denoted by *M*, follows a Poisson distribution with a mean of *m*Δ*t*. For each migration event, the frequencies of loci that change the GC follow a binomial distribution *B*(*lz_i_*, *ε*). Let *Y_h_* be the proportion of loci that transits among allelic GCs after a particular migration event. As several such events may occur over the chosen time interval under consideration there were no new mutations (*m* > *ul*), we consider *Y*_1_, *Y*_2_, … ,*Y_M_* to be the proportions of loci undergoing transition after a series of migration events, the properties of which are described by $E[Y_{h}]=z_{i}\varepsilon$ and $\mathrm{Var}[Y_{h}]=z_{i}\varepsilon (1-\varepsilon )/l$. $\Delta z_{i}=\sum_{h=1}^{M}Y_{h}$ is the frequency of loci that changes in terms of GC over the time interval Δ*t*, assuming that the extent of change in each event is rather small, and hence, *z_i_* can be approximated as a constant over this interval. Thus, Δ*z_i_* follows a compound Poisson distribution, allowing the mean and the variance to be calculated as shown in the electronic supplementary material, S3 Text.

Furthermore, a single migration event (e.g. from island A to B) may cause multiple loci to assume one of two different configurations. For example, assume that *z_i_* is the frequency of loci with the GC (A)(BC) and *z_j_* is that of the proportion with the GC (A)(B)(C). Then, a migration event from island A to B occurs at a rate *m*, following a Poisson distribution. The same migration event can change the numbers of loci with configuration (A)(BC) and those with configuration (A)(B)(C). Changes in *z_i_* and *z_j_* are positively correlated because both occur after the same successful migration event. To represent this partial correlation, we assume that Δ*z_i_* and Δ*z_j_* are linear combinations of stochastic terms, some of which are shared. By choosing the coefficients of such terms in a suitable manner, we can construct a simple SDE that has the means, variances, and covariance of the original process (electronic supplementary material, S3 Text).

Using such methods, we can construct a simple model simulating the stochastic processes associated with Δ*z_i_* (*i* = 1, 2, … ,5). Our method of calculating the impacts of migration among islands on the frequencies of loci in different GCs can be described as follows. First, we define the following notation to represent the frequency of transition from configuration *i* to configuration *j* caused by successful migration from island *α* to island *β* at rate *m_αβ_*:

2.11*a* $$f(\alpha \beta ,i\to j)=m_{\alpha \beta }z_{i}\varepsilon \Delta t+\sqrt{m_{\alpha \beta }}z_{i}\varepsilon \Delta W_{0}^{\alpha \beta }+\sqrt{\frac{m_{\alpha \beta }z_{i}\varepsilon (1-\varepsilon )}{l}}\Delta W_{i}^{\alpha \beta }.$$

Table 1 lists all possible transitions between GCs; six forms of migration between islands (each with three transitional GCs) are shown. In addition, table 1 includes two other transitions of GC, which we denote by *k* → *l* and *p* → *q*, caused by successful migration from island *α* to island *β*:

2.11*b* $$f(\alpha \beta ,k\to l)=m_{\alpha \beta }z_{k}\varepsilon \Delta t+\sqrt{m_{\alpha \beta }}z_{k}\varepsilon \Delta W_{0}^{\alpha \beta }+\sqrt{\frac{m_{\alpha \beta }z_{k}\varepsilon (1-\varepsilon )}{l}}\Delta W_{k}^{\alpha \beta }$$

and

2.11*c* $$f(\alpha \beta ,p\to q)=m_{\alpha \beta }z_{p}\varepsilon \Delta t+\sqrt{m_{\alpha \beta }}z_{p}\varepsilon \Delta W_{0}^{\alpha \beta }+\sqrt{\frac{m_{\alpha \beta }z_{p}\varepsilon (1-\varepsilon )}{l}}\Delta W_{p}^{\alpha \beta }.$$

Here, $\Delta W_{0}^{\alpha \beta }$, $\Delta W_{i}^{\alpha \beta }$, $\Delta W_{k}^{\alpha \beta }$ and$\Delta W_{p}^{\alpha \beta }$are independent stochastic variables with means of zero and variances Δ*t*.

**Table 1.** Changes in GCs caused by migration. (Six forms of migration are illustrated, with the subsequent changes in GCs. For example, the first row indicates migration from population A to B occurring at a rate *m*_AB_. This creates opportunities for changes in the GC from 2 to 4, 3 to 1 or 5 to 4. Loci with GCs 1 and 4 do not show changes upon such migration because populations A and B already contain the same fixed allele.)

origin of migration	destination of migration	rate of migration	change in GC		
A	B	*m*_AB_	2 → 4	3 → 1	5 → 4
B	A	*m*_BA_	2 → 1	3 → 4	5 → 4
A	C	*m*_AC_	2 → 3	4 → 1	5 → 3
C	A	*m*_CA_	2 → 1	4 → 3	5 → 3
B	C	*m*_BC_	3 → 2	4 → 1	5 → 2
C	B	*m*_CB_	3 → 1	4 → 2	5 → 2

Next, we derive the changes in the frequencies of loci with different GCs. As the mathematics are bulky, here we outline only the change in configuration (A)(BC) as an example:

2.12 $$\begin{array}{rl} {\left(\Delta z_{2}\right)}_{\mathrm{migration}} & =-f(AB,2\to 4)-f(BA,2\to 1)-f(AC,2\to 3)\\ & \;-f(CA,2\to 1)+f(BC,3\to 2)+f(BC,5\to 2)\\ & \;+f(CB,5\to 2)+f(CB,4\to 2). \end{array}$$

The first term on the right represents the rate of transition from configuration (A)(BC) to (C)(AB) caused by migration from A to B. The second term is the rate of transition from configuration (A)(BC) to (ABC) when migration from B to A is successful. These events reduce the number of loci with configuration (A)(BC). By contrast, the fourth term on the right of equation (2.12) represents the transition from (B)(AC) to (A)(BC) when migration from B to C is successful. The other terms can be interpreted in a similar manner. In the electronic supplementary material, S4 Text, we derive equations showing the changes in the other five GCs caused by migration.

## 3. Results

### 3.1. Dynamics of genetic distances in a two-island model

**Figure 2.**
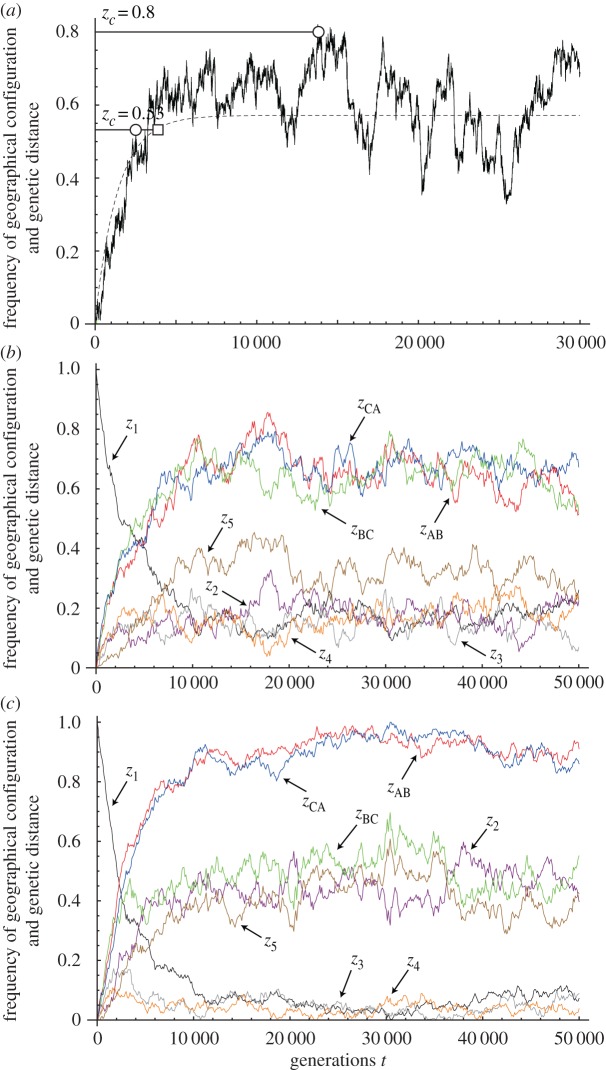
Dynamics of genetic distance and fractions of GC. The horizontal axis represents time expressed as a number of generations. (*a*) Evolutionary trajectory of genetic distance *z* between two islands. Initial condition satisfies *z* = 0. The black line is generated by SDE and the grey dashed line represents deterministic trajectory. The dynamics quickly converges to quasi-equilibrium. Two levels of speciation threshold *z_c_* = 0.53, 0.8 are shown. Open circles represent the points at which the trajectory calculated by SDE reaches each threshold. An open square is the point that the deterministic dynamics reaches the threshold (the value can never reach the higher threshold *z_c_* = 0.8). Because of stochasticities, dynamics simulated by SDE can reach a threshold that is higher than equilibrium. The parameters are *l* = 20, *u* = 0.0002, *m* = 0.015 and *ε* = 0.01. (*b*) The three lines in the upper portion of the figure indicate the proportions of incompatibility-controlling loci that differ between the two populations. By contrast, the five lines in the lower part of the figure are the frequencies of the number of loci of each configuration. See the text for definitions of the *z_i_* symbols and the GCs. Initially, all three islands share the same allele (*z*_1_ = 1 and *z*_2_ = *z*_3_ = … = *z*_5_ = 0). The migration rates between islands are equal, *m_αβ_* = 0.005, and the other parameters are *l* = 100, *u* = 0.0001 and *ε* = 0.01. (*c*) Dynamics of genetic differentiation among the three islands, which is as same as (*b*) except for the migration parameters. Here, we set migration rates *m*_AB_ = *m*_CA_ = 0.001 and *m*_BC_ = 0.01, respectively.

Figure 2*a* illustrates the trajectories of genetic distances *z* between two islands. The broken line indicates the deterministic dynamics, which are the same as the mean rate of change *M*(*z*), but includes no stochasticity (see Material and methods). This line increases smoothly to the asymptotic value z* = *u*/(*u* + *ε*); z* is the equilibrium of the corresponding deterministic model: d*z*/d*t* = *M*(*z*). Parameters are the number of incompatibility-controlling loci *l*, mutation rate per locus *u*, rate of successful migration event per generation *m* and impact of each migration *ε*. A product of *m* and *ε*, we hereafter call migration rate, is the average number of individuals that migrate from a population to another per generation. If z* is smaller than the threshold *z_c_*, the deterministic approximation predicts that speciation will never occur. However, because of stochastic fluctuations around equilibrium, *z* calculated by SDE becomes higher than z*, generating a *z_c_* within a finite period of time even when *z_c_* > z*. In such a situation, the magnitude of variance in genetic distance strongly impacts the time to speciation. Hence, the mean change in genetic distance is not sufficient for predicting the time to speciation. An accurate estimation of the variance of genetic distances must be determined.

### 3.2. Dynamics of geographical configurations in a three-island model

From the definition of the GC for each locus, (ABC) indicates that all three islands have the same allele. By contrast, (A)(B)(C) indicates that three islands have different alleles. Between these extremes, there are three intermediate configurations: (A)(BC) indicates that islands B and C have the same allele, but island A has a different allele. In a similar manner, (B)(CA) and (C)(AB) can exist. Let *z*_1_ be the fraction of loci of GC (ABC), and *z*_2_, *z*_3_, *z*_4_ and *z*_5_ the numbers of loci of status (A)(BC), (B)(CA), (C)(AB) and (A)(B)(C), respectively.

In figure 2*b*, we show the stochastic dynamics of GCs (*z*_1_, *z*_2_, … ,*z*_5_) and the fractions of incompatibility-controlling loci differing between two populations (*z*_AB_, *z*_BC_ and *z*_CA_). Under the defined initial conditions, the same allele is shared among the three populations for all loci, and *z*_1_ = 1, *z*_2_ = … = *z*_5_ = 0 and *z*_AB_ = *z*_BC_ = *z*_CA_ = 0. Over time, *z*_1_ decreases because different alleles become fixed in various populations and *z*_AB_, *z*_BC_ and *z*_CA_ become positive. Finally, all *z_i_* and *z_αβ_* converge towards their equilibrium values and continue to fluctuate around these values (cf. [48–51]).

In figure 2*c*, the dynamics of configurations assume that island A is isolated from the other islands (i.e. *m*_AB_ = *m*_CA_ = 0.001 and *m*_BC_ = 0.01). We can see that *z*_2_ is much larger than *z*_3_ and *z*_4_, indicating that *z*_AB_ and *z*_CA_ is generally larger than *z*_BC_. From this, we expect that a population on island A is more likely to become a separate species than populations on other islands.

### 3.3. Speciation event in three or more islands

In the two-island scenario, speciation occurs when the genetic distance exceeds a threshold value. Once this happens, migration between the two populations ceases and the genetic distance continues to increase. Hence, one can reasonably assume that the populations will never again become mixed. By contrast, if the model contains three or more islands, one population may have connection(s) via migration with other island(s), rendering it difficult to define exactly when speciation occurs. When the genetic distance between two populations exceeds a threshold value, genetic mixing via direct contact between individuals on the islands would cease. However, the populations may continue to belong to the same species if they are connected indirectly by a chain of migration bonds between islands, each of which has a genetic distance shorter than that of the threshold.

When the distances between both A and B, and A and C exceed the threshold, population A can be regarded as a separate species from populations B and C. In general, we define speciation as an event that occurs when an initially single species becomes split to reside on two clusters of islands, between which no migration occurs. Once this happens, mutation accumulation can be calculated with *m* = 0.

### 3.4. Average waiting time to evolution of two and three species

**Figure 3.**
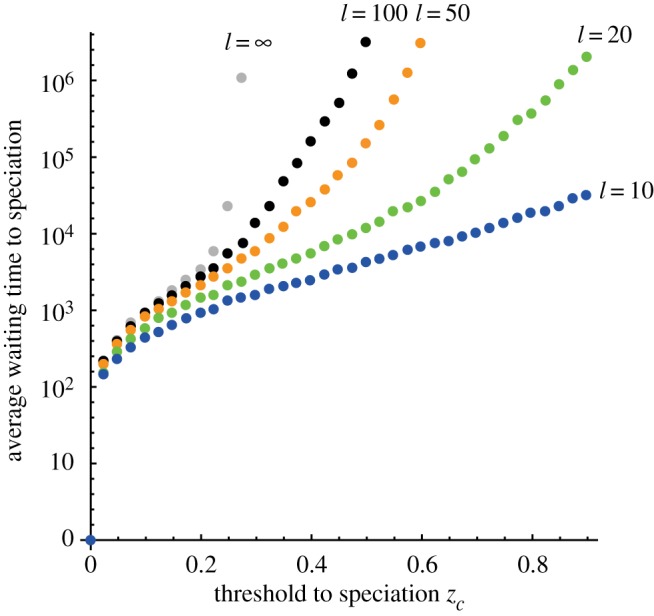
The average waiting times to speciation in a three-island model for different numbers of loci: *l* = 10,20,50,100 and ∞, as indicated by dot labels. The initial number of different loci was zero. The horizontal axis shows the thresholds to speciation, *z_c_*. Dots are the results from the SDE (average of 200 runs). The time to speciation for a finite *l* is much shorter than that for an infinitely large *l*. Other parameters are: *u* = 0.000075, *m_αβ_* = 0.01 and *ε* = 0.025.

Figure 3 shows the average waiting time to speciation in a three-island model, defined as the first occasion on which two of the three migration bonds become shut down. The waiting time monotonically increases with the threshold value. The figure shows the results for the different number of incompatibility-controlling loci *l* values, which were obtained by SDE (200 average for each plot). The finiteness of the number of loci *l* enhanced the variance in the genetic distance *z* and reduced the time to speciation. If we make the number of loci *l* infinitely large, the time to speciation becomes much longer than the value when *l* is finite. This is because the magnitudes of these stochasticities converge to 0 as *l* → ∞ (indicating that an infinitely large number of loci control the incompatibility).

**Figure 4.**
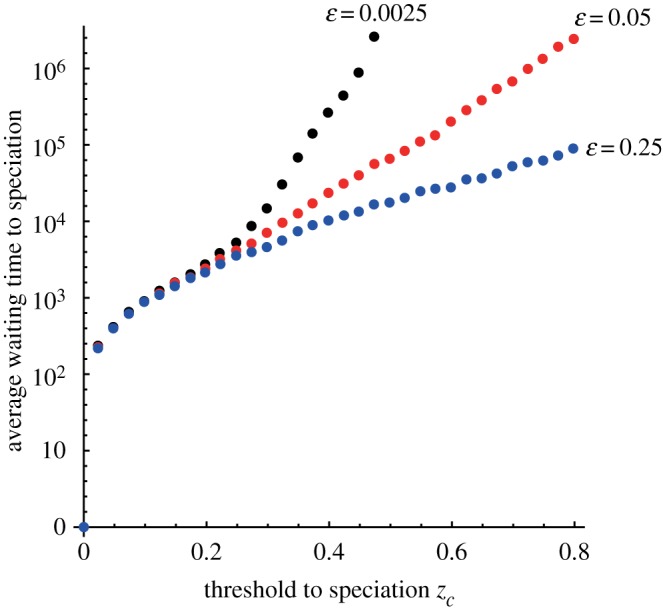
Average waiting time to speciation for different values of *m* and *ε* when their product is fixed as *mε* = 0.00025. Specifically, (*m*, *ε*) = (0.1, 0.0025), (0.005, 0.05) and (0.001, 0.25). The deterministic dynamics are the same, but the variance-generating rate increases with *ε*. The initial number of different loci was zero. The horizontal axis is *z_c_*, the threshold to speciation. Dots are the results from the SDE (average of 200 runs). The average time to speciation becomes shorter for a larger *ε* with *m* = 0.00025/*ε*. Other parameters are *l* = 100 and *u* = 0.000075.

The deterministic dynamics are specified by mutation rate *u* and migration rate *mε* only. Hence, the average waiting time to speciation obviously becomes longer when we set migration rate as a positive value. However, the magnitude of fluctuation is controlled by other parameters. For example, if the rate of successful migration events *m* decreases and the impact of each migration event *ε* increases while their product, *mε*, is unchanged, then the variance-generating rate given by equations (2.11*a*–*c*) (see Material and methods) is greater (note ε≪1 is assumed). Thus, the magnitude of fluctuation around the quasi-equilibrium increases, making the mean time to speciation considerably shorter. This effect is illustrated in figure 4.

**Figure 5.**
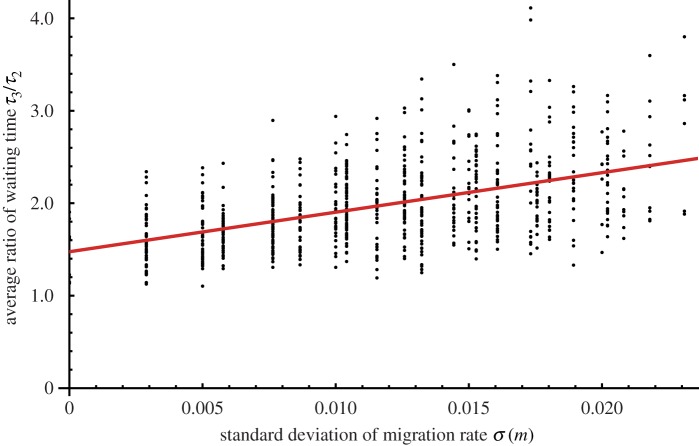
Average ratios of the waiting time to speciation of three and two species on three islands. The horizontal axis shows the standard deviations of migration rates among islands. Black dots represents the average ratios of waiting times, *τ*_3_/*τ*_2_, calculated by conducting 50 computer runs using stochastic differential equation (SDE) data for single sets of migration parameters. The red solid line is the linear regression of these dots. The migration rates were *m_αβ_* = 0,0.005, 0.010, … ,0.040, and we examined all combinations of the three migration types. The threshold fraction for speciation was *z_c_* = 0.3. The other parameters are *l* = 20, *u* = 0.000075 and *ε* = 0.01.

In the three-island model, a second speciation event occurs to create the third species when all three migration bonds are shut down. We can calculate the waiting times to two and three species, denoted by *τ*_2_ and *τ*_3_, respectively. If any migration rate *m_αβ_* increases, both *τ*_2_ and *τ*_3_ also increase. The ratio of the two waiting times *τ*_3_/*τ*_2_(≥1) indicates the relative lengths of these times and depends on a combination of migration rates. Figure 5 shows that the average ratio of the two waiting times increases with the standard deviation of the migration rate $\sigma (m)=\sqrt{\mathrm{Var}(m_{\alpha \beta })}$. The migration rates differ greatly when three migration bonds are considered, and the time to three species is much longer than the time to two species.

## 4. Discussion

We studied the average waiting time to speciation of a species living on multiple islands (or island-like habitats) between which migration occurs only infrequently. We assumed that the populations are monomorphic most of the time and that there is a threshold fraction of incompatibility loci controlling genetic mixing. The dynamics of the genetic distance among populations includes stochasticity from three sources: the timing of successful migration, the fixation of genes carried by migrants and the fixation of novel mutations.

Consider a pair of populations connected by a third population via migration. Migration events occurring through such bonds may either increase or decrease the genetic distance between the focal population pair, causing indirect effects of migration on the genetic distances. In addition, a single migration event between islands provides an opportunity for multiple loci to experience replacement of the resident allele by a migrant sequence. This causes correlation of the three distances between islands, making the variances and covariances between changes in distances difficult to evaluate. Note that the problem does not appear in the two-island model.

To overcome this problem, we introduced the novel concept of GC at each locus. This method can be used to evaluate whether different islands share the same allele. We developed the SDE method for tracing the dynamics of the fraction of loci exhibiting different GCs. The proportions of loci in different configurations can then be calculated using the SDE, from which we can reconstruct the fractions of incompatibility-controlling loci differing between two populations. In principle, the concept of GCs can be extended to the cases of *n* islands in general, although the mathematics becomes very difficult if *n* is large. There is often more than one set of GCs, all of which correspond to the same triplet of genetic distances. In the electronic supplementary material, S5 Text, we explain the correspondence between these configurations.

Figure 2*b* shows that the frequencies of different configurations and genetic distances fluctuate greatly, exhibiting strong asymmetry between islands, although the migration rates among islands are equal. The proportions of different GCs yield information not only on genetic distances among islands but also the proportions of unique alleles on any island at any given time.

Using the method introduced in this paper, we can analyse a system using SDEs, which is much faster than using the individual-based population genetic model. We could derive results similar to those derived for two islands as described by Yamaguchi & Iwasa [34]. First, the finiteness of the number of loci enhanced the variance in the genetic distance and reduced the waiting time to speciation (figure 3). Suppose that the number of loci becomes infinitely large while the threshold fraction *z_c_* remains unchanged. Then, two of the three sources of variance (fixation of genes carried by migrants and fixation of novel mutations) would be absent and the magnitude of fluctuation around the quasi-equilibrium would be reduced. This indicates that the low number of incompatibility-controlling loci should enhance the variation of population fluctuation around the quasi-equilibrium compared with the case of infinitely many loci. Many datasets from the genomic analysis would reveal the number of loci contributing to a reproductive isolation mechanism accurately in the near future. We can see that the low number of incompatibility-controlling loci has little effect on the results when the threshold is smaller than the equilibrium, but has a strong effect when the threshold is larger than equilibrium, as is the case for two islands [34].

Second, the increase in *ε* while keeping *mε* fixed has a similar effect as the low number of incompatibility-controlling loci (figure 4). The deterministic dynamics are specified by *u* and *mε* only, but the magnitude of fluctuation is controlled by its combination. For example, if the rate of migration events *m* decreases and the impact of each migration event *ε* increases with their product *mε* unchanged, then the variance-generating rate around the quasi-equilibrium increases. Thus, the magnitude of fluctuation around the quasi-equilibrium increases, making the mean time to speciation considerably shorter. This indicates that migrants arriving as a group and long periods without migration (these are realized at the same time) shorten the waiting time to speciation compared with the same number of migrants arriving individually, even if migration rates *mε* are the same. It might be interesting to compare phylogenetic diversity among taxa based on the relationship between migration rate and the number of immigrants at each migration event. These results for waiting time to speciation were derived in a two-island model [34], but the same results were confirmed for three islands when variance and covariance were handled properly.

In this study, we assumed that incompatibility between populations was absent when the genetic distance was less than a threshold value, but became fully established when the distance exceeded that of the threshold. However, a more realistic assumption is that the rate of successful migration decreases gradually as genetic distance increases. Immigrant viability decreases and hybrid sterility increases upon accumulation of mutations [52–54] and can promote speciation [3,55,56]. Another possible extension of our model is considering adaptation to a separate environment in different islands, which can be realized by modelling mutation rate including a selection coefficient. In addition, linkage and recombination among loci might also affect the speciation process by producing stochasticity including some correlations.

Although this paper did not compare the results obtained with two and three populations, Yamaguchi & Iwasa [33] compared average waiting time to speciation for up to 10 populations. The study suggested that an increase of the number of populations result in a longer waiting time to speciation. This is because variances in dynamics of genetic distance decreases with the number of populations though mean speed of the dynamics remains unchanged. However, the paper also suggested that the difference in waiting time to speciation between two- and three-island models is quite small. Thus, an extended research in this context is speciation dynamics in meta-population model with the concept of GC of allele sharing. In addition, genetic differentiation of ring species may provide a demonstration of how geographical differentiation on the species level can occur during ongoing gene flow [57]. The idea that two reproductively isolated populations are connected by a chain of intermediate populations corresponds to the case in which one of three genetic distances exceeds a threshold value in our three-island model. In this study, we regarded these populations as a single species. In order to elucidate the general dynamics of species diversity, a method of GCs for a larger number of islands must be developed.

The overall goal of our research on allopatric and parapatric speciation is to understand how speciation rates depend on geographical structure, particularly those involving varying numbers of islands that differ in terms of among-island migration rates. Thus, an important theme of future theoretical studies will be how speciation rates depend on various aspects of geographical structure, including differences in migration rates (figure 5), the centrality of some populations and the extent of transitivity in meta-populations. If multiple species are present, the speciation rate may depend on the number of other species coexisting in the system. In many theoretical models, exploring biodiversity patterns in community ecology, speciation rates are assumed to be constant within meta-populations and the emergence of a novel species is treated as a process similar to a point mutation (e.g. [58]). However, allopatric/parapatric speciation is in fact more consistent with fission speciation, in which a group of local populations becomes split from all other populations of the species. Combining the rate of allopatric/parapatric speciation and the rate of species extinction will provide a powerful theory for predicting biodiversity patterns.

In Yamaguchi & Iwasa [33,34,59], we studied recurrent parapatric (nearly allopatric) speciation when there were two islands. In the perfect absence of recurrent migration, no new species would be produced after populations in the two islands become separate species. By contrast, the number of species increases further if recurrent migration occurs between the two islands at a low and positive rate to result in a colonization event of immigrants after speciation. Recurrent migration between two islands that occurs at a very slow rate provides a simple mechanism to continue generating novel species. We concluded that an intermediate optimal rate of migration exists that realizes the fastest rate of species generation, which is consistent with the observation of molecular phylogeny of birds and organisms on an archipelago [60,61]. When the number of islands increases, combination of migration bonds between each island also increases rapidly. Thus, speciation as an event that occurs when an initially single species becomes split to reside on two clusters of islands might be difficult in a large number of islands. In this situation, evaluating stochasiticity of genetic dynamics might be necessary to calculate speciation dynamics. The study of recurrent species creation when there are three or more islands by using the GC method will be an important theme in future theoretical studies.

## Supplementary Material

The derivation of the formula

## Data accessibility

Datasets supporting this article are available as the electronic supplementary material, Texts (S1–S5). A simulation code written in C to produce data and a sample derived data for this paper are available from the Dryad Digital Repository: http://dx.doi.org/10.5061/dryad.mn65t [62].
