# Supplementary material for: Parapatric speciation in three islands: dynamics of geographical configuration of allele sharing
Source: R Soc Open Sci. 2017 Feb 22;4(2):160819. doi: 10.1098/rsos.160819 (PMC5367319; doi:10.1098/rsos.160819)
Supplement: The derivation of the formula [file rsos160819supp1.pdf]

Royal Society Open Science

Supplementary Texts (from S1 to S5)

Parapatric Speciation in Three Islands: Dynamics of Geographic Configuration of Allele Sharing

by

Ryo Yamaguchi[1], and Yoh Iwasa[1]

[1]*Department of Biology, Faculty of Science, Kyushu University, 744 Motooka, Nishi-ku, Fukuoka 819-0395, Japan.*

S1 Text:

Mutation and allelic replacement

From Eq. (1), we calculate the mean and the variance of the change in z caused by mutation and allelic replacement as follows:

$$E\left[(\Delta z)_A^{rep}\right] = u(1-z)\Delta t\,, \qquad \text{(S1.1a)}$$

$$Var\left[(\Delta z)_A^{rep}\right] = E\left[\left((\Delta z)_A^{rep}\right)^2\right] = \frac{u(1-z)}{l}\Delta t + O\left((\Delta t)^2\right), \qquad \text{(S1.1b)}$$

from which we have $M(z)=u(1-z)$ and $V(z)=u(1-z)/l$. Hence, we have formula Eq. (4), which has the same mean and variance as Eq. (1), but with different higher order moments. According to diffusion theory, the first and the second order moments determine the stochastic process, but moments higher than the second order do not affect the process in the limit of very low Δt. In a similar manner, we have

$$(\Delta z)_B^{rep} = u(1-z)\Delta t + \sqrt{\frac{u(1-z)}{l}} \bullet \Delta W_B^{rep}\,. \qquad \text{(S1.2)}$$

Migration and subsequent hybridization

From Eq. (2), we can derive

$$E\left[(\Delta z)_{AB}^{mig}\right] = -mz\varepsilon\Delta t\,, \qquad \text{(S1.3a)}$$

$$Var\left[(\Delta z)_{AB}^{mig}\right] = E\left[\left((\Delta z)_{AB}^{mig}\right)^2\right] = mz^2\varepsilon^2\Delta t + \frac{mz\varepsilon(1-\varepsilon)}{l}\Delta t + O\left((\Delta t)^2\right). \qquad \text{(S1.3b)}$$

The mean change in (S1.3a) is the decrease in z caused by migration, each reducing z on average by $z\varepsilon$. This migration event occurs in the Poisson process at rate m. The first term on the right-hand side of Eq. (S1.3b) indicates the variance caused by this Poisson process. The second term is the variance caused by the stochasticity of how many loci are taken over by the invaded alleles rather than by resident alleles. In Eq. (5) in the text, we show these two stochasticities separately using independent white noise.

For the migration occurring from island B to island A, we found a formula similar to Eq. (5):

$$(\Delta z)_{BA}^{mig} = -m\varepsilon z\Delta t + \sqrt{m}\varepsilon z \bullet \Delta W_{BA}^{mig} + \sqrt{\frac{mz\varepsilon(1-\varepsilon)}{l}} \bullet \Delta W_{BA}^{fix}\,, \qquad \text{(S1.4)}$$

S2 Text:

Example showing the need for tracing geographic configurations rather than the between-population genetic distances

We consider the following two cases:

Case 1: (ABC) holds for one-thirds of loci, and (A)(B)(C) holds for two-thirds of loci (l/3 and 2l/3 loci, respectively).

Case 2: Three geographic configurations, (A)(BC), (B)(CA), and (C)(AB) hold for one-third of loci (i.e. exactly l/3 loci).

Then, the genetic distances between the two islands are: $z_{AB} = z_{BC} = z_{CA} = 2/3$ for both cases.

Consider that a migration event occurs from island A to island B. This should provide an opportunity for each locus to experience corresponding changes in the geographic configuration. However, this may differ between cases:

Case 1: Loci of (ABC) remain unchanged after migration from A to B. Some loci of (A)(B)(C) may be transitioned to (C)(AB). Let k_3 be a variable following binomial distribution $B(2l/3, \varepsilon)$. After the migration event, (C)(AB) holds in k_3 loci and (A)(B)(C) holds in $2l/3 - k_3$ loci. Hence, we have

$$lz_{AB} = \frac{2l}{3} - k_3, \tag{S2.1a}$$

which leads to $E[lz_{AB}] = \frac{2l}{3}(1-\varepsilon)$ and $Var[lz_{AB}] = \frac{2l}{3}\varepsilon(1-\varepsilon)$. In contrast,

$$lz_{BC} = \frac{2l}{3}, \tag{S2.1b}$$

which leads to $E[lz_{BC}] = \frac{2l}{3}$ and $Var[lz_{BC}] = 0$.

Case 2: Loci of (C)(AB) remains unchanged. Loci of (A)(BC) or (B)(CA) may change. Let k_1 and k_2 be two independent variables following a binomial distribution $B(l/3, \varepsilon)$. Then, after migration, they produce (C)(AB) in k_1 loci, (A)(BC) in l/3-k_1 loci; (ABC) in k_2 loci and (B)(CA) in l/3-k_2 loci.

Hence, the number of loci at which islands A and B differ is

$$lz_{AB} = \frac{l}{3} - k_1 + \frac{l}{3} - k_2, \tag{S2.2a}$$

which leads to $E\left[lz_{AB}\right]=\frac{2l}{3}(1-\varepsilon)$ and $Var\left[lz_{AB}\right]=\frac{2l}{3}\varepsilon(1-\varepsilon)$. In a similar manner, the number of loci at which islands B and C differ is

$$lz_{BC}=\frac{l}{3}+k_1+\frac{l}{3}-k_2\ , \tag{S2.2b}$$

which leads to $E\left[lz_{AB}\right]=\frac{2l}{3}$ and $Var\left[lz_{AB}\right]=\frac{2l}{3}\varepsilon(1-\varepsilon)$.

Hence, in both case we have $E\left[z_{AB}\right]=\frac{2}{3}(1-\varepsilon)$, $Var\left[z_{AB}\right]=\frac{2}{3l}\varepsilon(1-\varepsilon)$, and $E\left[z_{BC}\right]=\frac{2}{3}$ hold. However, the variance of distance between island B and island C differ between the two cases:

$$Var\left[z_{BC}\right]=0\ , \qquad \text{for Case 1,}$$

$$Var\left[z_{BC}\right]=\frac{2}{3l}\varepsilon(1-\varepsilon)\ , \qquad \text{for Case 2.}$$

Then, concerning the mean value of distances, there is no difference between the two cases, but the variance of distance differs, indicating that without knowing the fractions of loci with different geographic configurations, we cannot predict the variance of the distances in the future. The time to speciation critically depends on the magnitude of variance of the distance fluctuation. This demonstrates the need for tracing the fraction of geographic configurations for each locus.

S3 Text:

Construct SDEs in a three-island model

Let Δz_i and Δz_j be the numbers of changes in z_i and z_j following successful migration events. In a similar manner, $\Delta z_j = \sum_{h=1}^{M} Y_h$ for Y_h $(h = 1,2,...)$ are independent and follow a different binomial distribution $B(lz_j, \varepsilon)$. We simulate these figures as follows:

$$\Delta z_i = A_i \Delta t + B_i \Delta W_1 + C_i \Delta W_3, \qquad \text{(S3.1a)}$$

$$\Delta z_j = A_j \Delta t + B_j \Delta W_2 + C_j \Delta W_3, \qquad \text{(S3.1b)}$$

where ΔW_i (i = 1, 2, 3) are independent stochastic variables with means of zero and variance Δt. Here A, B and C correspond to the different coefficients in eq. 11a of the main text. We choose the coefficients of the above expression in a manner to ensure that the equation was accurate in terms of all means, variances, and covariance.

From Eq. (S3.1a) and (S3.1b), we have

$$E[\Delta z_i] = A_i \Delta t, \quad E[\Delta z_j] = A_j \Delta t, \qquad \text{(S3.2a)} \quad \text{(S3.2b)}$$

$$Var[\Delta z_i] = \left(B_i^2 + C_i^2\right)\Delta t, \quad Var[\Delta z_j] = \left(B_j^2 + C_j^2\right)\Delta t, \qquad \text{(S3.2c)} \quad \text{(S3.2d)}$$

$$Cov[\Delta z_i, \Delta z_j] = C_i C_j \Delta t. \qquad \text{(S3.2e)}$$

Now, we consider the first and second moments of the process described above:

$$E[\Delta z_i] = m\Delta t \bullet z_i \varepsilon, \qquad \text{(S3.3a)}$$

$$E[\Delta z_j] = m\Delta t \bullet z_j \varepsilon, \qquad \text{(S3.3b)}$$

$$\begin{aligned} Var[\Delta z_i] &= E[M]\left(Var[Y_h] + E[Y_h]^2\right) \\ &= m\Delta t \bullet \left(\frac{z_i \varepsilon (1-\varepsilon)}{l} + (z_i \varepsilon)^2\right), \end{aligned} \qquad \text{(S3.3c)}$$

$$Var[\Delta z_j] = m\Delta t \bullet \left(\frac{z_j \varepsilon (1-\varepsilon)}{l} + (z_j \varepsilon)^2\right), \qquad \text{(S3.3d)}$$

where M is the expected number of migration events during time interval Δt. Thus, the covariance may be calculated as follows:

$$\begin{aligned}
Cov\left[\Delta z_i, \Delta z_j\right] &= E\left[\Delta z_i \Delta z_j\right] - E\left[\Delta z_i\right] E\left[\Delta z_j\right] \\
&= \sum_{k=0}^{\infty} \Pr\left[M = k\right] \left(\sum_{g=1}^{k} \left[Y_h\right] \sum_{h=1}^{k} \left[Y_h\right] \right) + o\left((\Delta t)^2 \right) \\
&= \sum_{k=0}^{\infty} \Pr\left[M = k\right] k^2 z_i z_j \varepsilon^2 + \cdots \\
&= \left\{ (m\Delta t)^2 + m\Delta t \right\} z_i z_j \varepsilon^2 + \cdots \\
&= m\Delta t \cdot z_i z_j \varepsilon^2 + \cdots .
\end{aligned}$$

Hence,

$$Cov\left(\Delta z_i, \Delta z_j\right) = m\Delta t \cdot z_i z_j \varepsilon^2 \,. \tag{S3.3e}$$

By incorporating the relationships shown below, Eq. (S3.4a–e) and Eq. (S3.3a–e) become mutually consistent:

$$A_i = m z_i \varepsilon \,, \quad A_j = m z_j \varepsilon \,, \qquad \text{(S3.4a)} \quad \text{(S3.4b)}$$

$$B_i = \sqrt{\frac{m z_i \varepsilon (1-\varepsilon)}{l}} \,, \quad B_j = \sqrt{\frac{m z_j \varepsilon (1-\varepsilon)}{l}} \,, \qquad \text{(S3.4c)} \quad \text{(S3.4d)}$$

$$C_i = \sqrt{m} \cdot z_i \varepsilon \,, \quad C_j = \sqrt{m} \cdot z_j \varepsilon \,. \qquad \text{(S3.4e)} \quad \text{(S3.4f)}$$

Adopting these values, scheme (S3.1) yields the same first moments (averages) and second moments (variances and covariance) of changes in z_i and z_j.

S4 Text:

Changes in geographic configurations caused by migration

The following terms express changes in the frequencies of loci exhibiting different geographic configurations:

$$\begin{aligned}(\Delta z_1)_{migration} &= f(AB,3\rightarrow 1)+f(BA,2\rightarrow 1)+f(AC,4\rightarrow 1)\\ &+f(CA,2\rightarrow 1)+f(BC,4\rightarrow 1)+f(CB,3\rightarrow 1)\end{aligned}, \quad \text{(S4.1a)}$$

$$\begin{aligned}(\Delta z_2)_{migration} &= -f(AB,2\rightarrow 4)-f(BA,2\rightarrow 1)-f(AC,2\rightarrow 3)\\ &-f(CA,2\rightarrow 1)+f(BC,3\rightarrow 2)+f(BC,5\rightarrow 2)\\ &+f(CB,5\rightarrow 2)+f(CB,4\rightarrow 2)\end{aligned}, \quad \text{(S4.1b)}$$

$$\begin{aligned}(\Delta z_3)_{migration} &= -f(AB,3\rightarrow 1)-f(BA,3\rightarrow 4)+f(AC,2\rightarrow 3)\\ &+f(CA,5\rightarrow 3)+f(CA,4\rightarrow 3)+f(CA,5\rightarrow 3)\\ &-f(BC,3\rightarrow 2)+f(CB,3\rightarrow 1)\end{aligned}, \quad \text{(S4.1c)}$$

$$\begin{aligned}(\Delta z_4)_{migration} &= f(AB,2\rightarrow 4)+f(AB,5\rightarrow 4)+f(BA,3\rightarrow 4)\\ &+f(BA,5\rightarrow 4)-f(AC,4\rightarrow 1)-f(CA,4\rightarrow 3)\\ &-f(BC,4\rightarrow 1)-f(CB,4\rightarrow 2)\end{aligned}, \quad \text{(S4.1d)}$$

$$\begin{aligned}(\Delta z_5)_{migration} &= -f(AB,5\rightarrow 4)-f(BA,5\rightarrow 4)-f(AC,5\rightarrow 3)\\ &-f(CA,5\rightarrow 3)-f(BC,5\rightarrow 2)-f(CB,5\rightarrow 2)\end{aligned}. \quad \text{(S4.1e)}$$

S5 Text:

Relationship between genetic distances between islands and geographic configurations

Suppose we are given triplet genetic distances (z_{AB}, z_{BC}, z_{CA}). In the following, we show that there is always at least one geographic distribution (GC) corresponding to (z_{AB}, z_{BC}, z_{CA}). It is often the case that there are multiple GCs. In addition, we can generate an explicit formula for all GCs corresponding to the given triplet (z_{AB}, z_{BC}, z_{CA}).

First, we note that the distances between the 3 populations satisfy the following:
$0 \le z_{AB} \le 1, 0 \le z_{BC} \le 1, 0 \le z_{CA} \le 1$, and in addition, they should satisfy:

$$z_{AB} \le z_{BC} + z_{CA}, \quad z_{BC} \le z_{CA} + z_{AB}, \quad z_{CA} \le z_{AB} + z_{BC} \qquad \text{(S5.1)}$$

These relationships are included in the axiom of distance, and distances between populations must satisfy these requirements. Based on this, we can prove the following proposition:

Proposition

We define the maximum of the three distances as $z_{\max} = \max[z_{AB}, z_{BC}, z_{CA}]$. z_1 must satisfy the following inequality.

$$\frac{2 - z_{AB} - z_{BC} - z_{BC}}{2} \le z_1 \le 1 - z_{\max} \qquad \text{(S5.2)}$$

We can prove that the rightmost side of Eq. (S5.2) is greater than the leftmost side. Then, we can express the following:

$$\begin{pmatrix} z_1 \\ z_2 \\ z_3 \\ z_4 \\ z_5 \end{pmatrix} = \begin{pmatrix} 0 \\ 1 - z_{BC} \\ 1 - z_{CA} \\ 1 - z_{AB} \\ z_{AB} + z_{BC} + z_{CA} - 2 \end{pmatrix} + z_1 \begin{pmatrix} 1 \\ -1 \\ -1 \\ -1 \\ 2 \end{pmatrix}. \qquad \text{(S5.3)}$$

All elements of a vector given by (S5.3) are either positive or zero and satisfy $\sum_{i=1}^{5} z_i = 1$.

Proof

We first define the similarity between populations as $s_{ij} = 1 - z_{ij}$. The relationship between this quantity and geographic distances between populations:

$$s_{AB} = 1 - z_{AB} = 1 - (z_2 + z_3 + z_5) = z_1 + z_4 \qquad \text{(S5.4a)}$$

In a similar manner, we can derive:

$$s_{BC} = z_1 + z_2 ,\ \ s_{CA} = z_1 + z_3 . \qquad \text{(S5.4b)}$$

Here, we define the minimum of similarities $s_{\min} = \min[s_{AB}, s_{BC}, s_{CA}]$. Since z_1 is the fraction of loci for which all populations share a common allele, this value should not exceed the minimum of the similarities. Hence, $z_1 \leq s_{\min}$ is maintained.

From Eq. (S5.4), $z_4 = s_{AB} - z_1$, $z_2 = s_{BC} - z_1$, $z_3 = s_{CA} - z_1$ hold. All of these values are nonnegative because of the choice of z_1.

$$\begin{aligned} z_5 &= 1 - z_1 - z_2 - z_3 - z_4 \\ &= 1 - z_1 - (s_{BC} - z_1) - (s_{CA} - z_1) - (s_{AB} - z_1) . \\ &= 1 - s_{AB} - s_{BC} - s_{CA} + 2z_1 \end{aligned} \qquad \text{(S5.5)}$$

Since this value is nonnegative, we have $z_1 \geq \frac{s_{AB} + s_{BC} + s_{CA} - 1}{2}$.

Hence, we have

$$\frac{s_{AB} + s_{BC} + s_{CA} - 1}{2} \leq z_1 \leq s_{\min} \quad . \qquad \text{(S5.6)}$$

Conversely, if we choose z_1 to satisfy Eq. (S5.6), the values of z_4, z_2, and z_3 as explained above must be nonnegative. Then, (S5.5) is also nonnegative. Then, the sum of these is equal to 1. Hence, vector (S5.3) corresponds to the distribution of geographic configurations.

However, we need to prove that the rightmost side is greater than the leftmost side. This can be conducted as follows:

The rightmost side minus the leftmost side of Eq. (S5.6) is equal to

$$s_{\min} - \frac{s_{AB} + s_{BC} + s_{CA} - 1}{2} = \frac{1}{2}\left(2s_{\min} - s_{AB} - s_{BC} - s_{CA} + 1\right)$$
$$= \frac{1}{2}\left(2\left(1 - z_{\max}\right) - \left(1 - z_{AB}\right) - \left(1 - z_{BC}\right) - \left(1 - z_{CA}\right) + 1\right)$$
$$= \frac{1}{2}\left(-2z_{\max} + z_{AB} + z_{BC} + z_{CA}\right)$$

Suppose $z_{\max} = z_{AB}$, the axiom of distance it is equal to $= \frac{1}{2}\left(-z_{AB} + z_{BC} + z_{CA}\right) \geq 0$. In a similar manner, we can prove nonnegativity when $z_{\max} = z_{BC}$ or when $z_{\max} = z_{CA}$. (End of the proof) .

Thus, if we choose z_1 to satisfy Eq. (S5.6), the distribution of geographic configurations can be written as:

$$\begin{pmatrix} z_1 \\ z_2 \\ z_3 \\ z_4 \\ z_5 \end{pmatrix} = \begin{pmatrix} 0 \\ s_{BC} \\ s_{CA} \\ s_{AB} \\ 1 - s_{AB} - s_{BC} - s_{CA} \end{pmatrix} + z_1 \begin{pmatrix} 1 \\ -1 \\ -1 \\ -1 \\ 2 \end{pmatrix}. \qquad \text{(S5.7)}$$

All elements of the vector given by (S5.7) are either positive or zero and satisfy $\sum_{i=1}^{5} z_i = 1$. Rewriting similarities by genetic distances makes (S5.6) and (S5.7) correspond to (S5.2) and (S5.3).
